# CT radiomic features of photodynamic priming in clinical pancreatic adenocarcinoma treatment

**DOI:** 10.1088/1361-6560/ac1458

**Published:** 2021-08-23

**Authors:** Phuong Vincent, Matthew E Maeder, Brady Hunt, Bryan Linn, Tiffany Mangels-Dick, Tayyaba Hasan, Kenneth K Wang, Brian W Pogue

**Affiliations:** 1 Thayer School of Engineering, Dartmouth College, Hanover NH 03755, United States of America; 2 Dartmouth-Hitchcock Department of Radiology, Lebanon NH 03756, United States of America; 3 Division of Gastroenterology and Hepatology, Mayo Clinic, Rochester, MN 55902, United States of America; 4 Wellman Center for Photomedicine, Massachusetts General Hospital, Harvard Medical School, Boston MA 02114, United States of America

**Keywords:** photodynamic therapy, radiomics, CT texture analysis, pancreatic cancer

## Abstract

Photodynamic therapy (PDT) offers localized focal ablation in unresectable pancreatic tumors while tissues surrounding the treatment volume experience a lower light dose, termed photodynamic priming (PDP). While PDP does not cause tissue damage, it has been demonstrated to promote vascular permeability, improve drug delivery, alleviate tumor cell density, and reduce desmoplasia and the resultant internal pressure in pre-clinical evaluation. Preclinical data supports PDP as a neoadjuvant therapy beneficial to subsequent chemotherapy or immunotherapy, yet it is challenging to quantify PDP effects in clinical treatment without additional imaging and testing. This study investigated the potential of radiomic analysis using CT scans acquired before and after PDT to identify areas experiencing PDT-induced necrosis as well as quantify PDP effects in the surrounding tissues. A total of 235 CT tumor slices from seven patients undergoing PDT for pancreatic tumors were examined. Radiomic features assessed included intensity metrics (CT number in Hounsfield Units) and texture analysis using several gray-level co-occurrence matrix (GLCM) parameters. Pre-treatment scans of tumor areas that resulted in PDT-induced necrosis showed statistically significant differences in intensity and texture-based features that could be used to predict the regions that did respond (paired t-test, response versus no response, *p* < 0.001). Evaluation of PDP effects on the surrounding tissues also demonstrated statistically significant differences, in tumor mean value, standard deviation, and GLCM parameters of contrast, dissimilarity and homogeneity (t-test, pre versus post, *p* < 0.001). Using leave-one-out cross validation, six intensity and texture-based features were combined into a support-vector machine model which demonstrated reliable prediction of treatment effects for six out of seven patients (ROC curve, AUC = 0.93). This study provides pilot evidence that texture features extracted from CT scans could be utilized as an effective clinical diagnostic prediction and assessment of PDT and PDP effects in pancreatic tumors. (clinical trial NCT03033225)

## 1. Introduction

Pancreatic ductal adenocarcinoma (PDAC) accounts for more than 90% of all pancreatic malignancies (Adamska *et al* 2017) with an abysmal 5 year survival rate of 8% (Kota *et al* 2017). Limited treatment options, with only 20% of the patients surgically resectable (Gillen *et al* 2010, Siegel *et al* 2013), have fueled a drive towards developing and advancing alternative PDAC treatments that combine ablative technologies with systemic therapies. Along with this effort, photodynamic therapy (PDT) has been studied as a promising localized and well-tolerated focal treatment option for solid tumors (Kostron 2010, Karakullukcu *et al* 2011, Yano *et al* 2012). A light-activated drug is systemically introduced into the body but only at the regions treated with light will reactive oxygen species be generated to cause cell death. Beyond focal treatment effects, recent studies in murine models have reported that a lower dose of PDT, termed ‘photodynamic priming’ (PDP), could prove beneficial as a neo-adjuvant method to prime or alter the tumors to enhance chemotherapy or immunotherapy (Canti *et al* 1994, Korbelik 1996, Snyder *et al* 2003, Perentes *et al* 2014). Pre-clinical data have showed that PDP effectively relieves the physical tumor drug delivery barriers by targeting protein expressions responsible for drug resistance as demonstrated by Huang *et al* (2016, 2018). PDP has also been used to modulate the tumor extracellular matrix which leads to lower tumor pressure, allowing for better distribution and efficacy of chemo drugs. This biological and physiological change as a result of PDP was demonstrated by Perentes *et al* and Obaid *et al* both of which reported a decrease in desmoplasia and tumor pressure leading to improved efficacy of subsequent chemotherapy (Perentes *et al* 2014, Obaid *et al* 2019). However, while PDT effects could be observed by identifying necrotic areas on a patient’s CT scan (Jermyn *et al* 2014), effects from low-dose PDP are more difficult to discern and often require *ex vivo* imaging techniques or further pathology staining, both of which are not clinically feasible with PDAC treatment. In this study, the CT scan alterations seen in clinical treatments were examined in a pilot cohort, to assess the potential of using tumor texture changes to predict and quantify PDP outcomes.

Radiomics has recently emerged as an effective method to obtain further information from clinical image data (Gillies *et al* 2016, Lambin *et al* 2017, Rizzo *et al* 2018). PET, CT and MRI are widely used to assist doctors in both diagnosis and therapy, yet only a limited amount of image data is extracted. For example, CT scans are acquired to identify tumor stages, but quantitative data reported remains on first-order parameters such as tumor size and average CT density value in hounsfield units (HU). Texture analysis with the goal of extracting further information indiscernible to simple inspection has offered a tool to provide quantitative data that are proven to be clinically valuable. In the field of pancreatic cancer, a few studies have been conducted using texture analysis to evaluate treatment effects (Chen *et al* 2017), predict survival outcomes (Cozzi *et al* 2019) or stratify different types of cystic lesions (Dalal *et al* 2020). Since PDAC has significant stromal components (Feig *et al* 2012, Rhim *et al* 2014, Neesse *et al* 2015) often leading to lack of perfusion and high heterogeneity, texture analysis is speculated to be of potential utility. Furthermore, biopsy is typically avoided in PDAC so diagnostic information from CT can be particularly valuable as CT imaging is perhaps the most widely used tool to assess tumor status.

**Figure 1.**
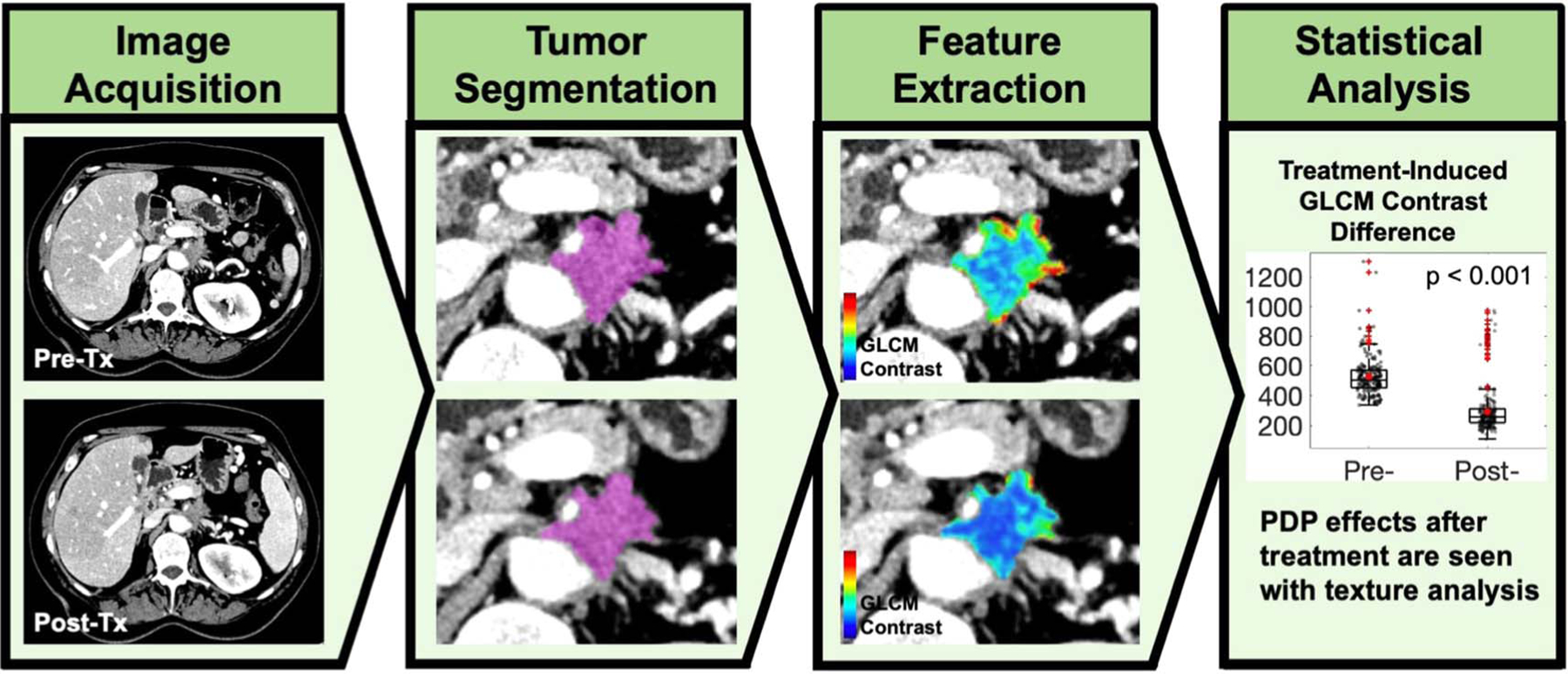
Overview of texture analysis in assessment of photodynamic therapy treatment on PDAC patients. Each patient received a pre-treatment and a post-treatment CT scan. Pancreatic tumor segmentation was performed, and texture analysis was carried out to extract underlying features that showed significant differences after the treatment.

This study was carried out with the objective of using texture analysis to examine CT scans before and after photodynamic light treatment to assess the underlying effects (figure 1). LIFEx software, a widely used Radiomics package (Nioche *et al* 2018), was used to segment regions of interest and perform texture analysis. The first aim of the study focused on comparing tumor regions that responded to light treatment with focal necrosis (termed PDT regions). The second part of the study was to analyze areas that did not show apparent necrosis but were suspected to be within the treated tumor (termed PDP regions). This second aim to employ texture analysis for evidence of PDP was done by comparing pre-treatment and post-treatment CT scans to identify differences that would show underappreciated texture-based changes in the images.

## 2. Methods

### 2.1. Patient population

Locally advanced PDAC patients were recruited at the Mayo Clinic (Rochester, MN) for the PDT trial. There were seven patients receiving treatment in this pilot study and their pre-treatment and post-treatment CT scans were used. All scans were fully anonymized prior to access and analysis. A summary of patient characteristics is provided in table 1. Most patients were in T3 stage with tumor site located at the pancreatic head. Patient inclusion and exclusion criteria along with treatment protocol are detailed in (Hanada *et al* 2021). The initial tumor volume range was 23 ± 17 cm^3^. The patients received a pre-treatment CT scan to identify the tumor site around one week before the treatment occurred (with the exception of Patient 06 whose pre-treatment scans were 20 d before). One hour before the light treatment, Visudyne (Novartis, East Hanover, NJ) at 0.4 mg kg^−1^ body weight was intravenously injected using a bolus infusion over 10 min. The 1 h time point and drug dosing were derived from Huggett *et al* aiming to induce both vascular and cellular damage (Huggett *et al* 2014). Photosensitizer selectivity is governed by the enhanced permeability retention (EPR) effect, yet EPR is severely impaired in PDAC tumors with limited blood vessels. While this phenomena is well-documented in preclinical studies (Samkoe *et al* 2013), there is no such data in humans because of the limited access to the pancreas and the fact that biopsy is generally avoided to prevent further metastases. Thus, treatment specificity is achieved via the localized light exposure. Light treatment with endoscopic ultrasound guidance was performed using a 690 nm light source (Model PSU-FC, Changchun New Industries Optoelectronics Technology Co. Ltd, Jilin, China) at40 J cm^−1^. The light fiber was a 1 cm diffuser with a radio-opaque marker at the tip. This fiber was inserted via the duodenum into the tumor and was visible under ultrasound. 48 h after the treatment, a post-treatment CT scan was acquired to evaluate the treatment effects.

### 2.2. Image acquisition and segmentation

All CT scans were acquired by a Siemens Force DS/129 scanner with the tube voltage in the range of 100–120 kVp, the tube current in between 97 and 166 mAs, the pixel spacing varying between 0.742 and 0.859 mm and the slice thickness of 1 mm. Non-ionic contrast media iohexol (Omnipaque 300 mg I ml^−1^, GE Healthcare Ireland, Cork, Ireland) with bolus tracking was used in the image acquisition process. Scans were acquired post-injection at 35 s for late arterial phase and 90 s for portal venous phase. Texture analysis was performed on the portal phase of the scans (Eilaghi *et al* 2017, Cheng *et al* 2019, Sandrasegaran *et al* 2019). Also, previous studies (Fave *et al* 2015, Mackin *et al* 2018) have reported that the tube current variation does not affect tissues with high level of texture such as tumorous tissues.

Pancreatic tumor segmentation was performed by a clinical radiologist from the Department of Radiology at Dartmouth–Hitchcock medical center (Lebanon, New Hampshire). The radiologist was informed that the CT scans were acquired for a PDT treatment at the pancreatic tumor site and received a 1 h training on how to perform ROI segmentation in the software LIFEx. Slice-by-slice delineation of tumor boundary was manually drawn on the tumors of all patients. The basis of tumor boundary delineation was determined based on the late arterial scans since they provided a better tumor margin visualization. Additionally, for patients that showed a treatment-induced necrosis, those areas were also specified. A trained graduate student then complete the ROI filling based on the specified tumor boundaries. Air, dense calcification and large blood vessels were excluded from the ROIs. The complete tumor ROIs were then reviewed by the radiologist before texture analysis.

### 2.3. Texture analysis

Texture analysis was performed using a software called LIFEx with built-in first and second-order texture features (Nioche *et al* 2018). The CT portal venous phase scans were first resampled to a voxel size of 1 mm, and absolute boundaries using the minimum and maximum CT numbers of the ROIs were used. 2D texture analysis was used with no binning. The ROIs’ CT density values in HU were referred to as ‘CT number’ in LIFEx.

To account for the effects of the patient-by-patient variations and evaluate any possible inconsistencies in the imaging acquisition process, a few normalization and control testing steps were performed. Physical tumor size change before and after light treatment was reported with no considerable changes to ensure a fair comparison between the baseline CT scan and post-treatment scans as ROI sizes could affect texture analysis. Recommended resampling to 1 mm voxel size was done on all scans which previously had a range of pixel spacing in between0.742 and 0.859 mm. The time differences between the two scans with respect to the nature of PDAC tumor progression were considered as mentioned in section 2.1 and the Discussion section. Contrast media concentration variation was tested by examining the liver and the spleen ROIs from the pre-treatment and post-treatment scans. Mean and standard deviation of CT numbers for these organs were reported in figure 4 and the changes before and after treatments were less than 7% for most patients (except for Patient 06). This average change (6.6 ± 2.6%) was considerably smaller than the average changes observed in the tumor regions (14 ± 7.3%), which confirmed that in the tumor regions, treatment-induced changes were dominant. Patient-by-patient variations were accounted for in the statistical analysis. receiver operating characteristic (ROC) analysis with leave-one-out cross validation and the generated ROC curve for combined classifiers provided evidence for PDP effects in most patients despite Patient 01 as an inherent outlier, which was discussed in the discussion section

### 2.4. Statistical analysis

A student’s t-test was used to compare the differences in means without the assumption of equal variance using the two-tail analysis with *α* = 0.05. Combined classifiers to classify pre-treatment versus post-treatment CT images were then developed using the statistically significant radiomic features. Radial basis function support vector machine (RBF-SVM) classifiers were optimized and evaluated using leave-one-patient-out cross validation. A custom Python script to perform model optimization and ROC analysis was developed using Scikit-Learn (Pedregosa Fabianpedregosa *et al* 2011). Briefly, the dataset was organized by CT image with the previously identified statistically significant radiomic features. Additional parameters were the patient ID which was assigned after anonymizing and randomizing patient data, and a binary label for pre versus post-treatment image. Iterating over the data by patient, RBF-SVM models were fit to all but the one patient withheld for testing. After fitting, the model was used to predict the binary label all the images of the test patient. The probability output for each image alongside the ground truth labels were used to calculate an ROC curve for each patient. An average ROC curve was calculated by taking the mean and standard deviation at point on the curve across all patients. Area under the curve was calculated for each patient with the mean and standard deviation across all patients also reported.

## 3. Results

### 3.1. Post-treatment CT scans show a reduction in mean and standard deviation of tumor region values, suggesting a decrease in tumor density

**Figure 2.**
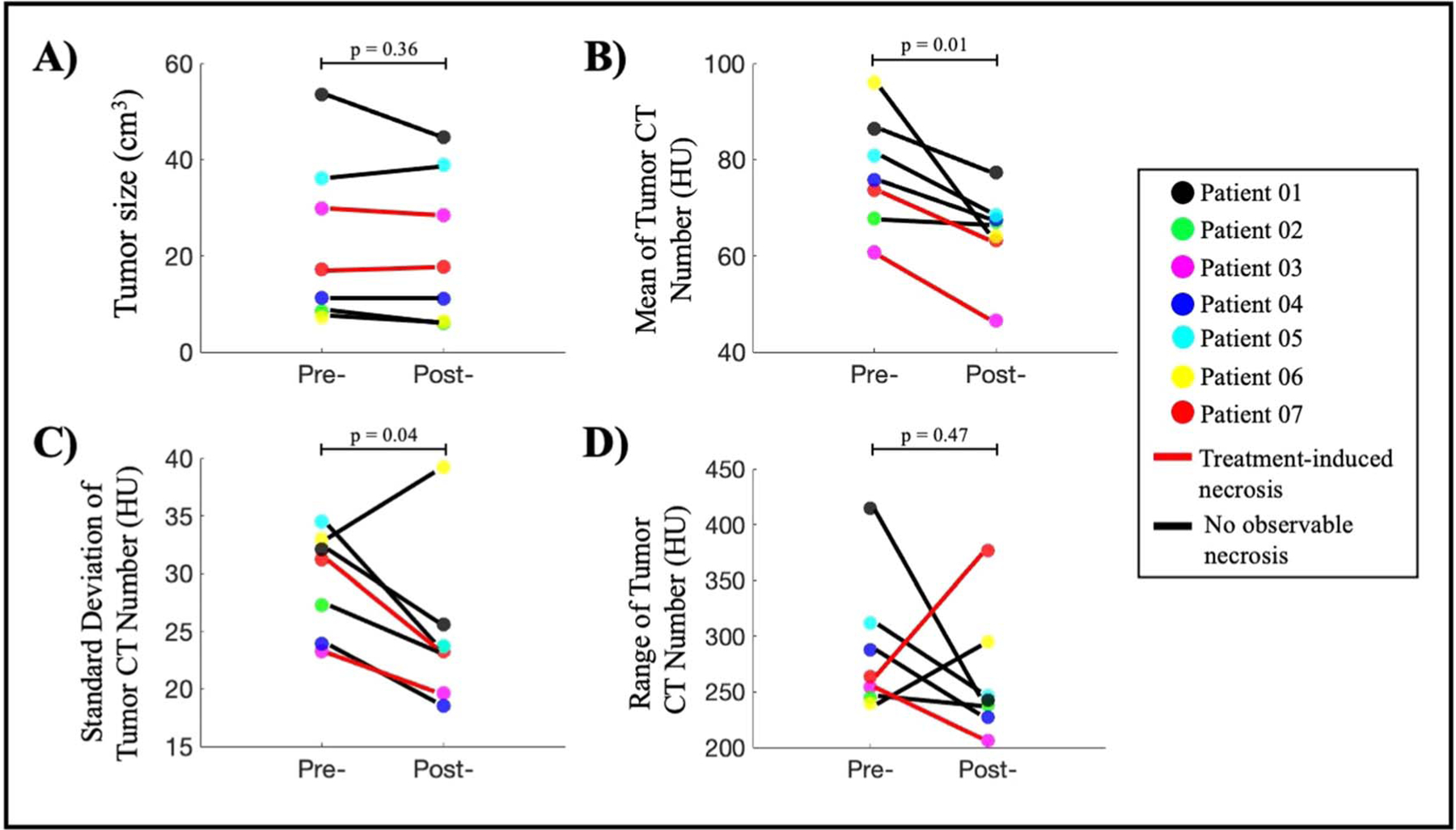
Overview of PDT-induced changes observed between pre- and post-treatment CT scans. (A) Tumor size measured from 3D segmentation of tumors shows no significant change (paired t-test, *p*-value = 0.36). (B) All patients show a reduction in mean tumor CT number, indicating the tumor density has decreased after PDT treatment (paired t-test, *p*-value = 0.01). (C) Six out of seven patients show a reduction in standard deviation of tumor CT number, suggesting that the intratumor variation has decreased after treatment (paired t-test, *p*-value = 0.04). (D) Range of CT number is decreased for five out of seven patients which indicates the reduction of intratumor variation after PD (paired t-test, *p*-value = 0.47).

Figure 2(A) reports tumor size as a result of slice-by-slice segmentation on pre- and post-treatment CT scans. The tumor size ranges from 8 to 54 cm^3^ with average pre-treatment tumor size of 23 ± 17 cm^3^ and average post-treatment of 22 ± 15 cm^3^, showing that there was not a significant change in size after treatment (paired t-test with *p*-value = 0.36). This finding is consistent with the patient’s radiology reports. The average CT numbers of the patient cohort pre- and post- treatment highlighted in figure 2(B) are 77 ± 11 HU and 64 ± 9 HU, respectively. Additionally, the standard deviation of CT numbers pre- and post- treatment in figure 2(C) are 29 ± 5 and 24 ± 5, indicating that the intratumoral changes were reduced after treatment. More interestingly, the average and standard deviation of CT number both show a statistically significant decrease after PDT treatment with the largest reduction of 33% in average HU and paired t-tests with *p*-values of 0.01 and 0.04, respectively (figures 2(B), (C)). Such reductions are good indicators that the tumor homogeneity is increased. The range of CT number maximum and minimum per tumor is also reduced in five out of seven patients, again suggesting the intratumor variation has been decreased after PDT treatment. The ROIs were drawn conservatively at the boundary to ensure only tumor tissues were included, therefore, the measurements of first-order parameters displayed in figure 2 are slightly lower than the actual values. However, this error is negligible since this segmentation approach is necessary to ensure texture analysis accuracy at the tumor margin. Most importantly, the overview of these first-order parameters shows a consistent trend suggesting a possible correlation between tumor density changes and PDT treatment effects, which could be quantified by texture analysis.

### 3.2. Texture analysis of pre-treatment CT scans can predict tumor areas that are pre-disposed to PDT induced necrosis response

**Figure 3.**
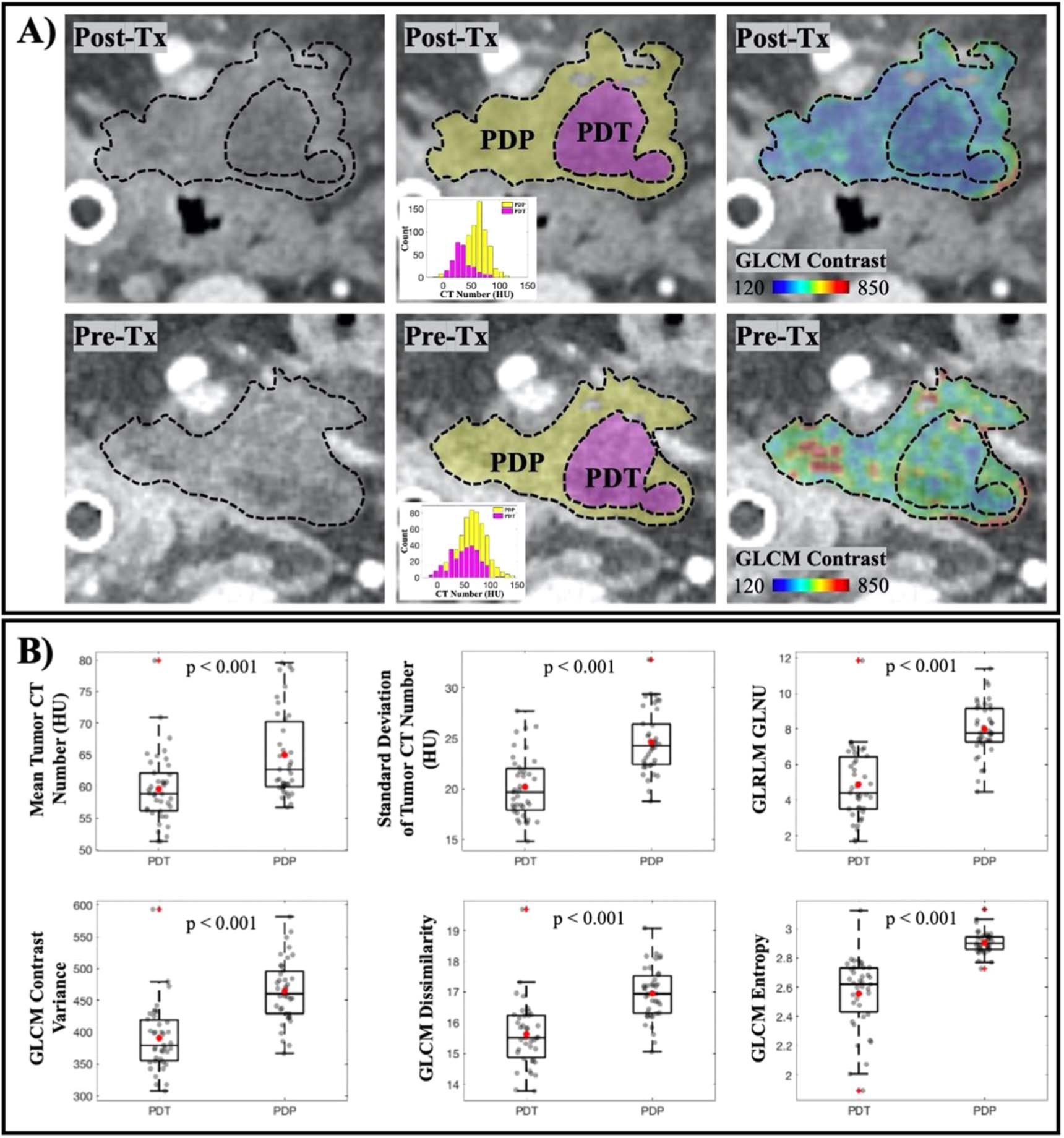
Treatment-induced necrosis areas could be predicted by texture analysis in the Pre-Tx scan. (A) Visualization of tumor in Post-Tx (top row) and Pre-Tx (bottom row) CT scans. Observable necrosis area (PDT) in pink and remaining tumor region (PDP) in yellow are delineated in Post-Tx scans, which were then mapped onto Pre-Tx scans for texture analysis. GLCM Contrast feature for both regions in the Pre-Tx scans is illustrated to show that necrosis occurred at the area with lower contrast. (B) Other first-order texture features that are found to be statistically significant when comparing the PDT and the PDP regions in Pre-Tx scans (*n* = 40 tumor slices).

From post-treatment CT scans, 40 tumor slices with a visible necrotic area as a result of PDT treatment were identified (figure 3(A)). For each tumor slice, manual segmentation performed by the radiologist separated the necrotic area in pink (PDT region) from the remaining of the tumor tissue in yellow (PDP region). These ROIs were then re-mapped back onto the pre-treatment CT scans in which texture analysis was performed. Results showed in figure 3(B) provided the texture features that were statistically significant between PDT and PDP regions. Those features included the tumor mean and standard deviation, GLCM contrast, dissimilarity and entropy, and GLRLM gray-level non-uniformity (paired t-test, *p* < 0.001).

### 3.3. Changes between pre-treatment and post-treatment CT scans in PDAC tumors indicate treatment effects, as referenced by other organ values

**Figure 4.**
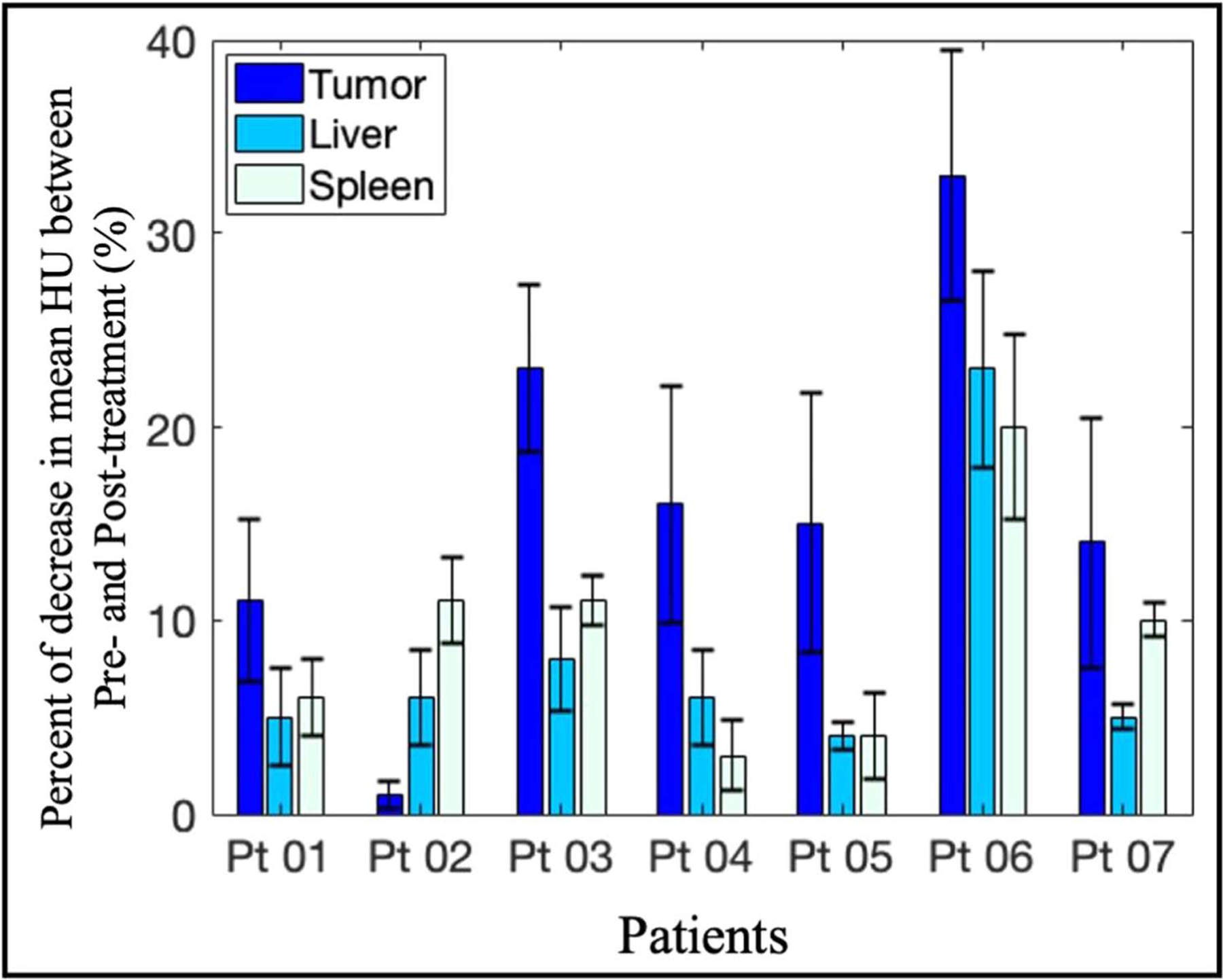
Percentage of changes after light treatment between pre-treatment and post-treatment scans in the PDP regions of tumors, as compared to the normal liver and spleen values. For each patient, the percentage change in CT mean was reported for the non-necrotic regions of PDAC tumor (14 ± 7.3%), liver (5.6 ± 1.3%) and spleen (7.7 ± 3.3%). Mean CT number decreased in all ROIs from all patients, with the tumor regions consistently expressing the biggest reduction in CT number. This observation suggested that there were treatment-induced effects on the tumor CT numbers across the patient cohort.

Besides visible necrotic effects observed in some patients, it was noticeable that in patients without tumor necrosis, there was a significant reduction in mean CT number between pre-treatment and post-treatment scans (figure 4). This decrease in CT number was shown to be significant when compared with that of neighboring normal organs such as liver and spleen. While changes in the liver and spleen across six out seven patients were5.6 ± 1.3% and 7.7 ± 3.3%, respectively, the decrease in non-necrotic (or PDP) tumor regions on average was 14 ± 7.3%. In some cases, the change in the tumor was more than doubled the changes in the liver and the spleen. It was important to realize that all ROIs from Patient 06 displayed more than 20% difference between the pre- and post-treatment scans, which was likely due to the 20 d elapsed before the acquisition of post-treatment scans as compared to within a week for other patients. Therefore, the differences observed in Patient 06 partially account for the rapid disease progression characteristic of PDAC. Overall, for most patients, the considerable difference in tumor CT number before and after treatment implies changes that are dominant by treatment effects and not just inherent variations in between different CT image acquisitions.

### 3.4. PDP effects could be quantified with texture analysis of pre- and post-treatment CT scans

**Figure 5.**
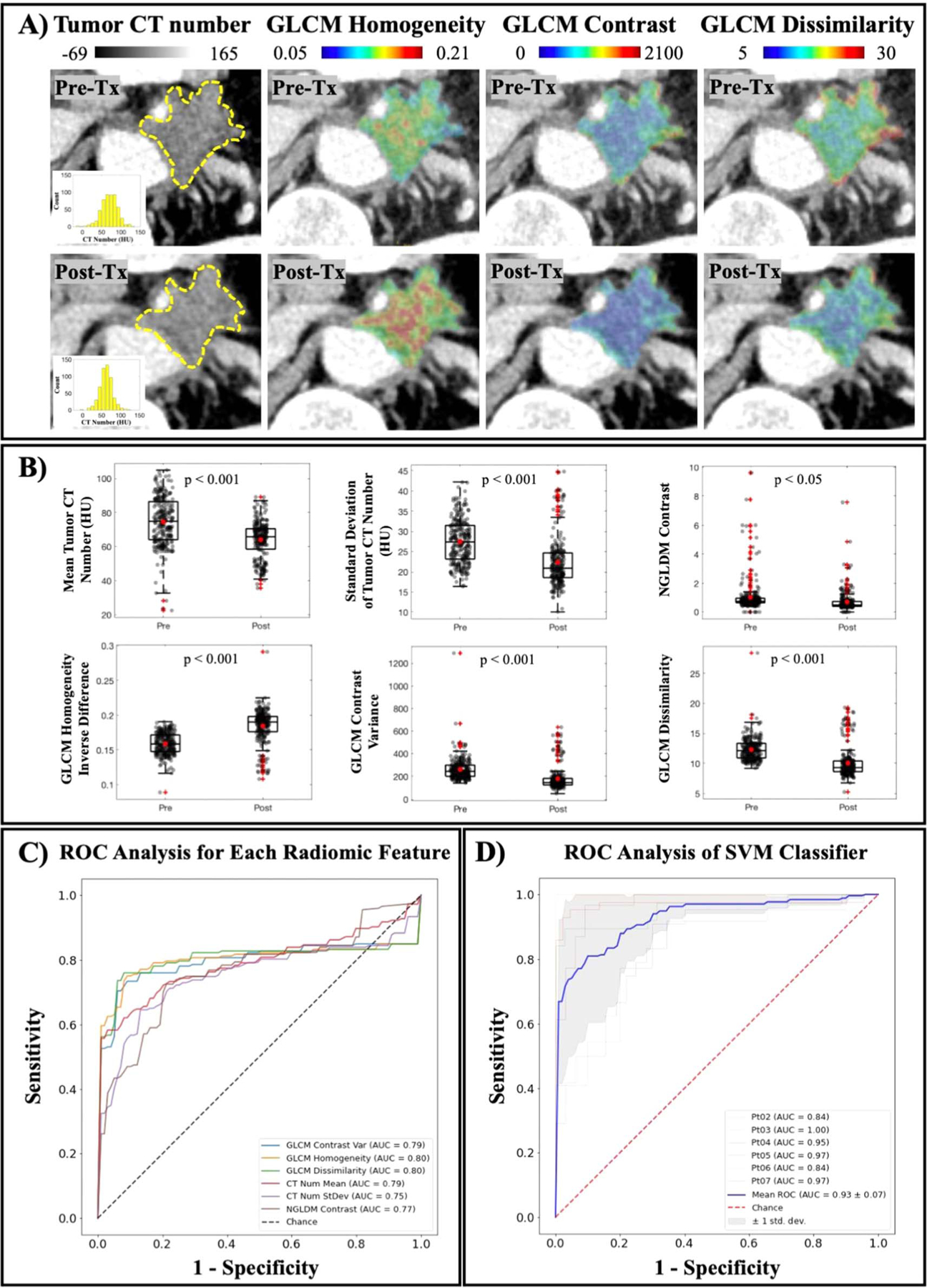
PDP effects after light treatment are seen with texture analysis. (A) A visualization of intratumor changes due to light treatment quantified by GLCM texture analysis. Means of tumor CT number before and after treatment are 74 ± 23 HU and 67 ± 17 HU, respectively. Tumor homogeneity is increased, while levels of contrast and dissimilarity are decreased, indicating a more uniform tumor attenuation profile. (B) Texture features that showed a statistical significance between Pre-Tx and Post-Tx scans are reported (*n* = 235 tumor slices) (C) Mean ROC curve for each reported feature calculated for all patients except for Patient 01, showing AUC values in the range of 0.75–0.80. (D) Mean ROC curve of combined classifiers using SVM model in blue yielded a better performance than any single classifier (AUC value is 0.93 ± 0.07). Leave-one-patient-out cross validation ROC curves are showed for all patients except Patient 01, and the shaded area indicated the ± 1 standard deviation of this cross validation.

Texture analysis was performed on non-necrotic tumor regions across all patients to study the underlying intratumoral changes as a result of PDT light treatment. Figure 5(A) illustrates the differences between pre-treatment and post-treatment CT scans. The first panel identified the ROIs in dotted yellow lines with corresponding histograms that showed a skewness to the left, indicating the decrease in mean CT number after treatment. A visualization of three GLCM parameters, homogeneity, contrast and dissimilarity, was also highlighted in figure 5(A) to showcase the differences between pre-treatment and post-treatments scans that are not appreciated in a conventional CT scan evaluation. Figure 5(B) reported the six most statistically significant texture features when compared between pre- and post-treatment scans with five out of six features showing a *p*-value of less than 0.001. In figure 5(C), an ROC curve with corresponding AUC value for each of the six texture features was generated. The ROC curves accounted for all patients but Patient 01. This patient was excluded from the classification process due to the large tumor volume, i.e. 54 cm^3^ compared to an average of 18 ± 12 cm^3^ of the remaining tumors. This considerably large tumor volume with the tumor’s diameter reaching 5 cm was convinced to not fully receive light treatment, therefore it was very unlikely to benefit from any PDP effects. Overall, figure 5(C) with overlaid ROC curves for all radiomic features as single classifiers showed that second-order GLCM features provided a better indicator of treatment effects as compared to tumor mean or standard deviation, emphasizing the need for texture analysis in this case.

Figure 5(D) displays the ROC curves for combining all six texture features into a classifier using the SVM model. Compared to using each feature as a single classifier with AUC value in the range of 0.75–0.80, a combination of all six yielded a better performance with a reported AUC value of 0.93 ± 0.07. Furthermore, an attempt to validate this classification method is also reported using the leave-one-patient-out cross validation approach as described in section 2.4. Each of the thinner ROC curves in figure 5(D) was generated to evaluate this classification method given the limited patient data. The shaded area displaying ± 1 standard deviation was plotted to visualize the effect of patient-by-patient variation on the classification. While Patient 01 was excluded as explained previously, the remaining patient cohort demonstrated a convincing classification with high AUC values in the range of 0.84–1.00. The ROC analysis in figure 5(D) provided evidence that identified texture features could be of great use when combined as a single classifier to evaluate the subtle intratumoral changes as a result of PDT treatment on non-necrotic tumor regions.

## 4. Discussion

PDAC aggressiveness combined with advanced stage at diagnosis has led to a desperate need for alternative therapeutic approaches. While research has focused on realizing new treatment options, it is equally important to identify effective approaches for accurate prediction of treatment outcomes and efficient assessment of treatment effects. In the case of PDT, evidence have showed that the most promising treatment effects are on the microscopic-level which requires additional imaging and/or special pathology staining of tumor samples. Low-dose PDT, also known as PDP, in preclinical data promises the relieving effects on the tumor microenvironment (Perentes *et al* 2014, Obaid *et al* 2019). Compared to the most visible effect of cell death via necrosis which could be visualized on a patient’s CT scan, the other benefits are more subtle, emphasizing the employment of further CT image analysis as validated in this study.

Texture analysis on pre-treatment CT scans focusing on the necrosis-induced tumor slices only showed that certain features could be used to predict tumor areas that are well-responded to PDT. Since analysis was only performed on pre-treatment images of tumor slices that later on expressed necrosis, each tumor slice contained a PDT and a PDP region to create paired data samples (figure 3(A)). Although the sample pool was limited (*n* = 40 tumor slices), paired samples assure that the differences reported are due only to the intrinsic tumor physiology. Figure 3(B) reported the six most statistically significant features when compared between PDT and PDP regions in pre-treatment scans. These features, namely tumor mean/stdev, GLCM contrast, dissimilarity and entropy, and GLRLM GLNU, all showed a *p*-value of less than 0.001. They also consistently support the idea that well-responded tumor areas are less dense, less disordered and more uniformed. While the limited data might not give a convincing statement on using these features as future treatment outcome identifiers, the data has indicated, for the first time, that tumor density and homogeneity directly affect PDT treatment outcome in clinical PDAC patients.

Data from figures 2 and 4 highlighted the intratumoral variation between pre-treatment and post-treatment CT scans as a result of PDP. Since all post-treatment CT scans were acquired 48 h after PDT, tumor shrinkage was not observed. Furthermore, due to the small sample size, it is currently not possible to draw any mechanistic interpretation of tumor volume changes. While necrotic tumor regions are reliable indicators of PDT treatment effects, PDP has only been explored recently and there is no correlation between tumor size reduction and treatment efficacy so far. The relatively constant tumor size across most patients ensures that comparison between pre- and post-treatment scans are consistent. More importantly, the significant reduction in patients’ tumor CT number corroborates with preclinical findings that PDT alleviates desmoplasia (Li *et al* 2018, Obaid*et al* 2019), an intrinsic characteristic of PDAC tumors responsible for elevated pressure and stiffness (Nieskoski *et al* 2017, Vincent *et al* 2020). Interestingly, the decrease in the standard deviation of tumor CT number for most patients (figure 2(C)) supports the hypothesis that tumor microenvironment heterogeneity, a physical barrier of intratumoral drug delivery efficiency (Chauhan *et al* 2011), become more uniformed after PDT treatment. This change in tumor heterogeneity was not only reflected by the decrease in CT number standard deviation, but it was further supported by GLCM texture features such as Homogeneity, Contrast and Dissimilarity reported in figure 5. While figure 5(A) provides a visualization of treatment-induced changes in tumor heterogeneity, the ROC analysis in figure 5(C) confirms that these second-order texture features performed better at identifying subtle intratumoral changes after light treatment. Moreover, combining all six of these features using SVM model greatly improved the classification performance with mean AUC value of 0.93 ± 0.07. The leave-one-patient-out cross validation was necessary given the limited patient cohort and the small patient-by-patient variation showed in figure 5(D) confirmed that a combination of these classifiers provided a great tool to evaluate PDP treatment effects. The exclusion of Patient 01 with considerably large tumor volume in the classification process was validated by the physical limitations of PDT, in which treatment efficacy relies on both the drug concentration and the amount of light exposure at the tumor site (Dougherty *et al* 1998). Furthermore, currently there is not a realistic and accurate way of identifying the PDP regions by simply looking at the CT images. Therefore, the combined classifiers as reported could be a reliable method to identify indiscernible PDP-affected zones for subsequent chemotherapy. While a larger patient cohort would be needed to realize this goal for future studies, this study has introduced an attractive approach for the use of radiomics analysis in pancreatic cancer treatment.

## 5. Conclusion

This study has provided early evidence to support the use of texture analysis on CT scans in predicting treatment outcomes and assessing therapeutic effects on PDAC patients undergoing PDT. In this limited patient cohort with different cancer stages, the study shows that for all stages, well-responded tumor regions were less dense, had lower average CT number and more uniform. These observations align with preclinical data and are classified via GLCM and GLRLM texture features. More importantly, the indiscernible, microscopic-level effects of photodynamic priming (PDP effects) on alleviating tumor heterogeneity have been visualized and quantified by comparing pre- and post-treatment CT scans. Besides noticeable reductions in tumor CT number and its standard deviation, second-order GLCM features provided better classifiers, demonstrating for the first time with clinical data that better PDT outcomes occurred at regions with lower tumor density and higher homogeneity.

## Figures and Tables

**Table 1.** Summary of patient characteristics.

					Tumor volume (cm^3^)		Tumor attenuation (HU)	
Patient	Age (year)	Sex	Disease state	Tumor site	Pre-Tx	Post-Tx	Pre-Tx	Post-Tx
1	65	M	T3	Pancreatic head, tail, and neck	54	45	86 ± 32	77 ± 26
2	53	M	T2	Pancreatic head	8	6	68 ± 27	67 ± 24
3	73	F	T3	Pancreatic head	30	28	61 ± 23	47 ± 20
4	57	F	T3	Pancreatic body and tail	11	11	76 ± 24	68 ± 19
5	63	F	T3	Pancreatic head	36	39	81 ± 35	69 ± 24
6	62	M	T1	Pancreatic head	7	6	96 ± 33	64 ± 39
7	75	M	T3	Pancreatic neck	17	18	74 ± 31	63 ± 23
